# The Differences between Blast-Induced and Sports-Related Brain Injuries

**DOI:** 10.3389/fneur.2013.00119

**Published:** 2013-08-14

**Authors:** Yun Chen, Wei Huang, Shlomi Constantini

**Affiliations:** ^1^BrightstarTech, Inc.Clarksburg, MD, USA; ^2^Uniformed Services University of the Health SciencesBethesda, MD, USA; ^3^Department of Pediatric Neurosurgery, Dana Children’s Hospital, Tel-Aviv Medical Center, Tel Aviv UniversityTel Aviv, Israel

Both blast-induced traumatic brain injury (TBI) and sports-related concussion are occasionally considered to have similar injury mechanisms by which repeated head impacts cause chronic traumatic encephalopathy (CTE). Patients with the two conditions experience comparable CTE symptoms, such as headache, light-headedness, memory loss, confusion, attention deficits, difficulty balancing, aggression, anxiety, depression, etc. (1). Another common feature is tau protein deposition around cerebral blood vessels in the frontal cortex (2, 3). However, it should be noted that these symptoms are non-specific, and can be the results of many different brain insults such as TBI, stroke, chemically induced neurotoxicity, infection by pathogenic microbes (HIV virus, bacteria, etc.), brain tumors, and neurodegenerative diseases (Parkinson’s, Alzheimer’s, ALS, Huntingtons, etc.). Abnormal or hyper-phosphorylated tau protein deposition is also non-specific and represents only disruption and dysfunction of microtubules in neuronal cells that are experiencing progressive death and degeneration. Therefore, having similar mental symptoms and tau protein deposition in the same regions of the brain does not conclude that the same basic injury mechanism existed in both blast-induced and sports-related brain injuries.

## Injury Mechanism and Neuropathophysiology

Concussion is one of the most common sports-related injuries, which involves approximately 270,000 cases each year in USA, mostly occurring in football, ice hockey, soccer, wrestling, and basketball players (4, 5). Sports-related concussion is commonly caused by a direct impact to the head, face, neck, or other parts of the body in contact or collision sports, such as a collision with another player, a fall to the ground, or being hit by balls and bats. It occurs as a result of sudden acceleration/deceleration or rotational forces imparted to the brain (4, 6). Sudden linear acceleration/deceleration forces to the head are known to be a mechanism for closed head injury, which cause discrete, focal lesions that affect only certain areas of the brain (the point of direct impact and at the site directly opposite the point of impact) and often occur as in sporting activities, motor vehicle accidents, accidental falls, and assaults (7). Sudden rotational or angular movement of the head can cause shear strains resulting in neural damage in the brain. Patients with sports-related concussion generally have a history of prior concussion or head impact in sports activities, but the possibility of a concussion cannot be ruled out in the absence of the history of direct impact to the head.

In contra-distinction, a blast shock wave cannot cause a linear acceleration or rotational (angular) acceleration injury to the head, because sudden linear acceleration (whiplash) or angular rotation (jolting) of the head is most likely to cause a head injury concomitant with a cervical spine injury or an acute injury to the cervical spine. Until now, no soldiers with a blast-induced TBI have been found to sustain a concomitant cervical spine injury. The major mechanism of blast TBI may involve damage to the blood-brain barrier (BBB) and tiny cerebral blood vessels, which are caused by the blood surge moving quickly through large blood vessels to the brain from the torso (8). When a blast shock wave acts on the human body, the surrounding overpressure causes a sudden, transient (1–2 ms) increase in atmospheric pressure on the organs and the cardiovascular system. Elevated overall pressure on the cardiovascular system will rapidly raise blood pressure to cause a rapid physical movement or displacement of blood (a volumetric blood surge) (9). Because human skull can resist compression of a blast shock wave to avoid a sudden increase in intracranial pressure, the cranial cavity should be a relatively low-pressure area when a blast shock wave acts on the whole human body. Therefore, the volumetric blood surge will certainly move through blood vessels to the low-pressure cranial cavity from the high-pressure ventral body cavity (thoracic cavity and abdominal cavity). It will dramatically increase cerebral perfusion pressure and cause damage to both tiny cerebral blood vessels and the BBB in the global brain. Large-scale cerebrovascular insults and the BBB damage will trigger secondary neuronal damage (10).

Pathophysiological changes of sports-related concussion are very similar to that of TBI and stroke, which are characterized by impaired neurotransmission, abnormal regulation of ion channels, ionic imbalance, deregulated cellular energy metabolism, and decreased cerebral blood flow (11). Following concussion, disruption, and increased permeability of neuronal cell membranes cause extracellular potassium accumulation and excitatory amino acid (glutamate) release (12). Extracellular accumulation of potassium ions results in neuronal suppression through neuronal cell membrane depolarization. To restore normal neuronal function, sodium-potassium pump is activated, and cellular metabolic activities increase, which will increase both glucose and oxygen consumption in the brain and cause cerebral hyperglycolysis (4, 12, 13). These pathophysiological changes are usually observed in the area bordering the injured site (the impact site or opposite side of the skull), and not in entire brain.

The initial pathophysiological changes following blast-induced TBI are described as the pepper-spray pattern – diffuse cerebral edema, hyperemia, vasospasm, and hemorrhage in entire brain (14). Cerebral edema, hyperemia, hemorrhage, and vasospasm might be the results of large-scale tiny cerebrovascular insults and BBB damage that occur globally throughout the brain (8), which will further trigger a pathophysiological cascade leading to progressive neuronal cell death, neural loss, and axonal degeneration in the brain. The pathophysiological cascade has been defined as secondary neuronal damage. Secondary neuronal damage induces excitotoxicity, inflammation, ionic imbalance, oxidative stress, apoptosis, diffuse axonal injury, and neurodegeneration in the injured brain, and ultimately leads to neuronal cell death, loss, and degeneration.

## Tau Protein

Because abnormal or phosphorylated tau protein deposition was observed around cerebral blood vessels in the frontal cortex of both veterans exposed repeatedly to blasts and football players with repetitive concussive injury, tau protein was suggested as a biomarker of both blast-induced and sports-related brain injuries (2, 3). Tau protein is a highly soluble microtubule-associated protein (MAP) that interacts with tubulin to strengthen microtubules (the neural tubes in the axons of neurons) and promote tubulin assembly into microtubules. It is mainly found in neurons in the central nervous system (CNS), but can be expressed at very low levels in CNS astrocytes and oligodendrocytes (15). Tau protein is critical to maintaining normal functioning of CNS, which provides axonal microtubule assembly, stabilization, and flexibility, and also involves neurite outgrowth and axonal transport through its isoforms and phosphorylation (16).

Phosphorylation of tau protein is the predominant regulatory mechanism of its activity. Some kinases (GSK3β, cdk5, PKA, FYN, PKN, CaMKII, etc.) and phosphatases (PP2A, PP2B, etc.) are involved in the tau phosphorylation process and work in concert for tau protein to function correctly (17–19). Abnormal or hyper-phosphorylated tau protein can result in the self-assembly of tangles of paired helical filaments and straight filaments, which are involved in the development of Alzheimer’s disease and other diseases (such as TBI, Pick’s disease, corticobasal degeneration, frontotemporal dementia, and supranuclear palsy) (20). For example, when PKN is over-activated, it will cause hyper-phosphorylation of the tau protein, making the soluble protein become extremely insoluble aggregates entangled in microtubules and block neural signal transmission in neurons as the walls of the microtubules collapse (21). However, abnormal or hyper-phosphorylated tau protein may only represent disruption and dysfunction of microtubules in the neurons that are suffering secondary neuronal damage, and not the initial pathogenesis leading to progressive neuronal cell death and degeneration. In other words, abnormal tau protein found in the brains of both veterans and football players does not mean that blast-induced TBI has the same injury mechanisms as sports-related concussion.

The increased tau protein level was generally observed in the brain extracellular fluid, CSF, and serum of the patients with severe TBI (22–26), suggesting that tau protein can be considered as a biomarker of TBI. However, NFL players who had a history of repeated head injuries and showed significant levels of tau protein in their brains did not experience CTE symptoms (mood symptoms and cognitive deficits) (27). Phosphorylation of the tau protein can be affected by many biological components (kinases, phosphatases, etc.) involved regulation of tau phosphorylation. Any changes in these biological components will certainly result in hyper-phosphorylation of the tau protein, thus causing abnormal tau protein deposition. Therefore, the increased level of tau protein in the brain tissue, CSF, or blood is not a specific and accurate biomarker of TBI and CTE. Just as Dr. Kevin Crutchfield, the neurologist at the Comprehensive Sports Concussion Program of Sinai Hospital of Baltimore, MD, USA, said: “Tau can be a marker of the presence of disease but may not be a biomarker of disease activity. Placing too much emphasis on a marker of disease as a true biomarker without years of clinical disease correlation may lead to treating a finding without clinical relevance” (28).

If tau protein can be used as an indicator of blast-induced and sports-related brain injuries, one can consider neuronal cell death as a hallmark of both concussion injury and HIV infection because neuronal cell death/loss in the frontal cortex have been found in both cases of concussion injury (29) and HIV infection (30). However, no one, in fact, believes that concussion injury and HIV infection have the same pathogenesis or injury mechanism, even though neuronal cell death/loss is observed in the same region (frontal cortex) of the brain in both the conditions. For the same reason, tau protein cannot be used to specifically diagnose both blast-induced and sports-related brain injuries.

## Chronic Traumatic Encephalopathy

Chronic traumatic encephalopathy is a syndrome of global brain dysfunction which is caused by repetitive concussions or head injury. CTE, previously called dementia pugilistica (DP), was initially found in professional boxing athletes (31), and has been commonly found now in professional athletes who suffered multiple head trauma, such as football, ice hockey, soccer, wrestling, and basketball players (32). CTE is a progressive degenerative disease in the brain which generally appears years or decades after head trauma. It is typically difficult to detect CTE by using neuroimaging techniques (i.e., CT and MRI), because progressive neuronal cell death and degeneration can only be seen under a microscope after death. CTE is marked by the signs and symptoms of altered mental status such as memory loss, aggression, confusion, attention deficits, poor judgment, aggression, anxiety, depression, etc. (1). However, the altered mental status is a non-specific clinical manifestation that can be frequently observed in the patients with various brain insults. The neuropsychiatric signs and symptoms that are seen in the CTE patients also occur in many other brain insults (33).

In the cases of sports-related concussion, repeated head impacts are only the initial insult to the brain. Much of the damage done to the brain does not typically occur at the time of initial insult and does not result directly from the initial insult itself. The initial insult will trigger secondary neuronal damage. Secondary neuronal damage can possibly continue in the hours, days, weeks, or months following the initial insults. It is an indirect consequence of the initial insults and a major contributor to subsequent neuropsychiatric impairments. Secondary neuronal damage in some specific regions of the brain (such as the hippocampus, amygdale, thalamus, piriform cortex, and cortex) may be largely responsible for these chronic neuropsychiatric disorders. Cerebral edema, inflammation, calcium influx, and apoptosis program have been identified as the critical contributors to secondary neuronal damage in various brain insults (33). CTE is only a spectrum of neuropsychiatric and neurological disorders induced by secondary neuronal damage in the brain after the pathophysiological cascade is triggered by sports-related concussion or repeated head trauma. Secondary neuronal damage is actively involved not only in the development of CTE, but also in the onset of neurological and psychological disorders resulting from blast-induced TBI and other brain insults (8).

## Conclusion

The initial mechanism of blast-induced TBI is different from that of sports-related concussion (Table 1). However, both the two conditions can trigger a comparable pathophysiological cascade (i.e., secondary neuronal damage), thus leading to progressive neuronal cell death, neural loss, and axonal degeneration in the brain. Secondary neuronal damage should be the major contributor to ultimate neurologic and psychological impairments following various brain insults including blast-induced TBI and sports-related concussion. CTE is a result of secondary neuronal damage triggered by sports-related concussion. It is similar to long-term neuropsychiatric and neurological consequences of blast-induced TBI. This may be the reason why blast-induced TBI and sports-related concussion are considered to have the same injury mechanisms. Abnormal tau protein deposition in some specific regions of the brain is only a marker of the presence of neuronal cell death, loss or degeneration, and cannot be used to specifically diagnose blast-induced TBI and sports-related head trauma.

**Table 1:** **Comparison of blast-induced TBI with sports-related concussion**.

	Sports-related concussion	Blast-induced TBI
Cause of injury	Sudden acceleration/deceleration or rotational forces to the head	Blast shock waves
Mechanism of injury	Discrete, focal brain damage caused by direct impact forces	Large-scale tiny cerebrovascular insults and BBB damage caused by blood surge moving quickly to the brain from the torso
Pathophysiology	Similar to non-blast TBI and stroke (impaired neurotransmission, ionic imbalance, deregulated cellular energy metabolism, and decreased cerebral blood flow)	Pepper-spray pattern – diffuse cerebral edema, hyperemia, vasospasm, and hemorrhage
Abnormal tau protein deposition	Occurs in the injured area of the brain in a non-specific manner	Occurs in the injured area of the brain in a non-specific manner
Secondary neuronal damage	Triggered by the initial insult – mechanical force-induced brain damage	Triggered by the initial insult – blood surge-induced tiny cerebrovascular insults and BBB damage
Long-term clinical consequences	Altered mental status, CTE	Chronic neuropsychiatric disorders that are similar to CTE
